# Comparison of laser therapy and ozonated water on gingival inflammation in orthodontic patients with fixed appliances

**Published:** 2021-09-27

**Authors:** V. Kala Vani Sandra, Ziauddhin Shaik, Geetika Simhadri, Lakshmikar Reddy Obili Govindu Gari, Bindu Priya Siddamreddy, Naveen Makem

**Affiliations:** ^1^Department of Orthodontics and Dentofacial Orthopedics, C.K.S. Teja Institute of Dental Sciences and Research, Chadalawada Nagar, Renigunta Road, Tirupathi, Andhra Pradesh, India; ^2^Department of Orthodontics and Dentofacial Orthopedics, Coorg Institute of Dental Sciences, Kodagu, Coorg District, Virajpet, Karnataka, India; ^3^Department of Orthodontics and Dentofacial Orthopedics, Anil Neerukonda Institute of Dental Sciences, Sangivalasa, Bheemunipatnam, Visakhapatnam, Andhra Pradesh, India; ^4^Department of Orthodontics and Dentofacial Orthopedics, SJM Dental College and Hospital, P.B. Road, Chitradurga, Karnataka, India

**Keywords:** fixed appliance, gingival crevicular fluid, gingival inflammation, monocyte chemoattractant protein, laser therapy, ozone water

## Abstract

**Background:**

There is an increased prevalence of oral ailments such as dental caries, gingivitis, and periodontitis when orthodontic therapy is administered. Poor oral hygiene in conjunction with the placement of fixed orthodontic appliances is considered a significant factor in raising accumulation of bacterial plaque and its associated inflammatory response.

**Aim:**

The present study aimed to evaluate and compare laser therapy with subgingival irrigation using ozonated water on gingivitis in patients undergoing fixed orthodontic treatment and to ascertain the presence of the inflammatory marker monocyte chemoattractant protein (MCP-1) in gingival crevicular fluid (GCF).

**Methods:**

This study was a double-blind clinical study in which a split-mouth design was applied in 30 subjects for 28 days that received fixed orthodontic therapy. In each subject, the upper right quadrant (control side) was irrigated with 0.01 mg/L ozonated water and the upper left quadrant (experimental side) was irradiated with a diode laser on day 0 (baseline) and day 7. The patients were recalled on days 7, 14, and 28 and clinical parameters were recorded at each visit. Biochemical evaluation of gingival inflammation with MCP-1 levels in GCF was obtained at the baseline (day 0) and on day 28.

**Results:**

A statistically significant (p < 0.05) reduction of all clinical parameters and MCP-1 activity in GCF was observed on both sides. Low-level laser irradiation showed a significant reduction of clinical parameters and MCP-1 activity compared to subgingival ozone irrigation.

**Conclusions:**

Laser therapy can be considered a more effective method than subgingival ozone irrigation in patients undergoing fixed orthodontic treatment as it showed a consistent improvement in gingival inflammation.

**Relevance for patients:**

Fixed orthodontic appliances make oral hygiene maintenance difficult and results in gingivitis. Adjunctive oral hygiene procedures such as subgingival irrigation with ozonated water or laser irradiation are beneficial. The data from this study suggest that irrigation with ozonated water or laser irradiation reduces gingival inflammation, with laser therapy being more effective.

## 1. Introduction

Fixed orthodontic treatment is the preferred and most common method of treating malocclusion. However, fixed dental appliances may complicate optimal oral hygiene resulting in the accumulation of dental plaque and gingival inflammation leading to gingivitis and its progression to periodontitis [1]. In the early 1900s sodium bicarbonate was used to treat periodontal inflammation [2]. In 1913, hydrogen peroxide was shown to decrease plaque formation [3]. Several *in vivo* studies have demonstrated that sanguinarine can reduce gingivitis and plaque growth in the absence of mechanical plaque removal [4]. An essential factor in the use of sodium bicarbonate and hydrogen peroxide in periodontal treatment is their delivery to subgingival areas. To be effective these substances must reach the subgingival plaque. Various means can be used to administer therapeutic agents to the periodontal tissues; they include the toothbrush, toothpick, dental floss, oral irrigator, wedge-shaped wooden toothpick, rubber tip, interdental brush, mouth rinse, tray, ultrasonic instrument, and professional subgingival application with a curette [2].

In a study by Babay and Jesser, single subgingival irrigation of sanguinarine or saline on orthodontically banded first molars in adolescent patients effectively reduced the inflammation of interdental papilla with the best results favoring the saline group [5]. An alternative approach to conventional antimicrobial or antiseptic agents is by altering the subgingival microenvironment, which has been shown to be highly anaerobic with prevailing low oxygen tension. It was found that ozonated water (0.5 – 4 mg/L) was highly effective in the killing of both Gram-positive and Gram-negative microorganisms [6]. A study conducted by Ramzy *et al*. in 2005 concluded that there was a significant improvement in oral hygiene achieved with ozone irrigation [7]. From a study conducted in 2010 the investigators concluded that there was a marked reduction in gingival inflammation when ozonated water was used compared to chlorhexidine solution [8]. Other similar studies in orthodontic patients report that subgingival irrigation with ozonated water could effectively reduce gingival inflammation in orthodontic patients [9-11].

Laser is an acronym for light amplification by stimulated emission of radiation [12]. Laser treatment is known to coagulate blood vessels, seal lymphatics, and sterilize wounds during ablation while maintaining a clear and clean surgical field [13]. A laser can stimulate mitochondrial activity which results in biomodulation of inflammatory processes by mitigating prostaglandin E_2_ release; hence, they are recommended in the treatment of gingival inflammation [14,15]. A study conducted by Pejcic *et al*. showed low-level laser as an adjunct oral treatment resulted in the reduction of tissue inflammation and which was reflected in histopathological changes of the gingival tissue [16,17].

Chemokines have significant pro-inflammatory effects and are often associated with periodontal tissue destruction and the stimulation of bone resorption and induction of tissue damage. Monocyte chemoattractant protein-1 (MCP-1) is a protein classified as a chemokine secreted by various cell types including macrophages, fibroblasts, epithelial cells, and endothelial cells. MCP-1 plays an essential role in the pathogenesis of various inflammatory diseases and conditions such as periodontitis, multiple myeloma, Sjogren syndrome, and rheumatoid arthritis. Thus, MCP-1 could have clinical utility as a screening tool for discriminating the various stages of periodontal disease [18]. Until now studies were conducted that focused on evaluating the anti-inflammatory effect of either ozone or low-level laser in reducing gingival inflammation; however, there were no studies comparing their clinical efficiency. Since MCP-1 levels in gingival crevicular fluid (GCF) are a reliable marker predicting gingival inflammation, correlation of levels of MCP-1 with clinical parameters is desirable to ascertain treatment efficacy.

The aim of this investigation was to compare the efficacy of subgingival ozone water irrigation with low-level laser therapy and clinical parameters to determine their clinical efficacy in mitigating gingivitis in patients receiving fixed orthodontic therapy.

## 2. Materials and Methods

In a single-center prospective study, a split-mouth model was used to compare ozone water irrigation versus laser irradiation as adjunct oral hygiene treatment against gingivitis. Patients undergoing fixed orthodontic treatment were elidable for participation. Written informed consent was taken from each patient enrolled in this study and ethical clearance for the study was received from the Institutional (C.K.S. Teja Institute of Dental Sciences and Research) ethical committee.

### 2.1. Study participants

A total of 30 patients aged between 15 and 25 years who received active orthodontic treatment for at least 4 and 8 months and having at least 24 natural teeth (excluding 3^rd^ molars) were selected for this study. Enrolled subjects should neither have severe periodontitis nor rampant caries. Exclusion criteria were relevant medical history or use of any medication affecting the gingival health and using any removable prosthesis or other oral appliances. Patients are advised to continue regular oral hygiene habits, that is, brushing twice daily for a minimum of 2 min are continued in the same manner without any modification. The patients were recalled on the days 7, 14, and 28 for data acquisition.

### 2.2. Experimental design

A split-mouth design was planned and in each subject the upper right quadrant (control side) was irrigated with 0.01 mg/L ozonated water (Kent Ozone Dental Jet TY- 820, Kent Ro. Systems Ltd., Noida, India) and the upper left quadrant (experimental side) was irradiated with a 10 W diode laser (Zolar Photon Plus, Zolar Technology and Mfg. Co., Inc., *Mississauga*, ON, Canada). The period planned for the study was 28 days and the patients were recalled on days 7, 14, and 28.

### 2.3. Collection of clinical parameters

All the clinical parameters included in this study were recorded by a single examiner who was blinded to the treatment condition. Clinical parameters including plaque index (PI), gingival index (GI) and gingival bleeding index (GBI) were recorded on day 0 (baseline) and subsequently on days 7, 14, and 28. The clinical procedure started by making sure the patient was sitting comfortably in an upright position on a dental examination chair and sufficient illumination in the area of interest was provided during the examination. Then using a sterile mouth mirror and a William’s graduated periodontal probe, a periodontal status is recorded. On both the experimental and control sides, before and after the application of laser therapy and ozonated water, GCF samples were collected also at baseline (day 0) and on day 28. To reduce the possibility of contamination by pooled saliva from the mandibular region, the maxillary teeth were selected for data acquisition.

### 2.4. GCF sampling procedure

To collect GCF 1 – 3 mL calibrated volumetric microcapillary pipettes (Sigma-Aldrich Chemical Co., St. Louis, MO, USA) were used. To prevent contamination and blocking of the microcapillary pipette during collection of GCF supragingival plaque was removed carefully without touching the gingival margin. The tip of the pipette was placed extracrevicularly to prevent the collection of stimulated GCF. From each test site, 2mL (standard volume) of unstimulated GCF was collected. Test sites with a lack of standard volume of GCF and those pipettes contaminated with blood and saliva were excluded from the study. Later for the biochemical analysis, samples were stored in a refrigerator at −20°C at SVIMS, Dept. of virology, Tirupati.

### 2.5. Ozone irrigation

Irrigation with 0.01 mg/L ozonated water was achieved by using a modified needle attached to an irrigation device, Kent Ozone Dental Jet TY- 820 (Kent Ro. Systems Ltd., Noida, India), on the maxillary right quadrant. A single pulsating stream of ozonated water was released from the nozzle that could be adjusted for different speeds and pressures ranging from 350 to 500 kPa and an ozone output of 0.082 mg/h at a noise output of <70 dB with water outflow of ≥450 mL. A 22-gauge blunt needle was bent and attached to the tip of the nozzle to facilitate subgingival ozone water irrigation. As per the study design, irrigation was performed on day 0 (baseline) and on day 7 for 3 min per session.

### 2.6. Laser irradiation

As per the split-mouth design, laser irradiation using Zolar Photon Plus 10 W soft-tissue diode laser was administered on the left maxillary quadrant. The laser beam was placed at a vertical distance of 2 mm from the gingival surface and at a right angle to the gingival surface. For the anti-inflammatory effects, the following parameters were power density of 100 Mw/cm^2^, energy density of 18 J/cm^2^ with 3-min field exposure per session with irradiation set to continuous mode. The laser was applied at baseline (day 0) and on day 7.

### 2.7. ELISA procedure

MCP-1 presence was determined using an assay based on a sandwich ELISA protocol. On the ELISA plate, the capture antibody was coated and a coating stabilizer was used to stabilize it. To the wells, standards and samples containing antigen were added and incubated. Antigen and antibody complexes were formed during incubation. Biotin-labeled tracer antibody conjugated with streptavidin to horseradish peroxidase (HRP) (Thermo Fisher Scientific, Waltham, MA, USA) was used to detect the complexes. Tetramethylbenzidine (TMB) substrate was added which gave color that could be monitored and analyzed under a wavelength of 450 nm by an ELISA reader. A standard curve was used to estimate the concentrations of MCP-1 in the tested samples.

### 2.8. Statistical analysis

Using G*Power analysis (version 3.1.9.6; Heinrich-Heine-Universität Düsseldorf, Düsseldorf, Germany) sample size calculation was obtained with an estimated effect size of 0.71, an alpha error of 5% and a power of 95%, resulting in 28 (n = 14 per group). Hence, a sample size of 30 with 15 in each group was taken. To compare the data between test and control samples at various durations, statistical data were subjected to an independent t-test. They were also subjected to a Pearson correlation test which could correlate between clinical and biochemical parameters. Finally, an ANOVA test was used to analyze the differences among group means. For all statistical assessments, p < 0.05 was considered statistically significant.

## 3. Results

Intergroup comparisons of clinical parameters and MCP-1 across the 4 time points are presented in Table 1. Tables 2 and 3 present ANOVA analysis for laser irradiation and ozone water irrigation, respectively. Pearson’s correlation analyses are presented in Tables 4 and 5 for the control versus experimental sides for day 0 and day 28, respectively.

**Table 1:** Intergroup comparison of clinical parameters and MCP-1 levels at different intervals

Parameter	No. of samples	Day	Laser side		Ozone water side		p-value
	
			Mean	SD	Mean	SD	
GI	30	0^th^ day	2.140	0.148	2.067	0.204	0.116
PI	30		2.177	0.175	2.103	0.219	0.158
GBI	30		13.500	2.838	12.430	3.340	0.188
MCP-1	30		38.693	2.433	39.563	2.625	0.188
GI	30	7^th^ day	1.359	0.232	1.843	0.107	0.000**
PI	30		1.567	0.275	1.863	0.122	0.000**
GBI	30		8.830	2.842	11.570	2.956	0.001**
GI	30	14^th^ day	1.200	0.108	1.663	0.177	0.000**
PI	30		1.197	0.177	1.673	0.198	0.000**
GBI	30		4.500	2.418	10.070	3.362	0.000**
GI	30	28^th^ day	0.567	0.152	1.037	0.188	0.000**
PI	30		0.680	0.122	0.990	0.163	0.000**
GBI	30		0.930	1.388	5.600	3.169	0.000**
MCP-1	30		16.607	3.039	22.083	2.559	0.000**

** Significant; ANOVA test for the laser group, that is, on the experimental side, revealed that a statistically significant difference exists for all the parameters

**Table 2:** ANOVA for laser irradiation datasets

	Mean value	F value	p-value
GI			
Between sides	12.55781	456.2561	0.000**
Within sides	0.027524		
PI			
Between sides	11.90633	312.6154	0.000**
Within sides	0.038086		
GBI			
Between sides	886.5194	148.3857	0.000**
Within sides	5.974425		
MCP-1			
Between sides	7317.313	965.307	0.000**
Within sides	439.6573		

** Significant; ANOVA on the control side showed a statistically significant difference for clinical parameters and MCP-1 enzyme levels

**Table 3:** ANOVA for ozone water irrigated datasets

	Mean value	F value	p-value
GI			
Between sides	5.873194	195.792	0.000**
Within sides	0.03		
PI			
Between sides	6.86942	213.92	0.000**
Within sides	0.03211		
GBI			
Between sides	277.122	26.8826	0.000**
Within sides	10.3086		
MCP-1			
Between sides	4583.256	683.3799	0.000**
Within sides	388.9913		

** Significant

**Table 4:** Results of Pearson correlation test between MCP-1 and clinical parameters on control and experimental sides on day 0

Clinical parameter	Laser side				Ozone water irrigated side			
	
	GI	PI	GBI	MCP-1	GI	PI	GBI	MCP-1
GI								
Correlation	1.000	0.144	0.189	0.317	1.000	0.075	−0.011	0.458*
p-value	.	0.449	0.316	0.088	.	0.694	0.954	0.011
PI								
Correlation	0.144	1.000	0.010	0.197	0.075	1.000	0.053	0.292
p-value	0.449	.	0.957	0.298	0.694	.	0.780	0.118
GBI								
Correlation	0.189	0.010	1.000	−0.296	−0.011	0.053	1.000	−0.201
p-value	0.316	0.957	.	0.112	0.954	0.780	.	0.287
MCP-1								
Correlation	0.317	0.197	−0.296	1.000	0.458*	0.292	−0.201	1.000
p-value	0.088	0.298	0.112	.	0.011	0.118	0.287	.

* Significant

**Table 5:** Results of Pearson correlation test between MCP-1 and clinical parameters on control and experimental sides on day 28

Clinical parameter	Laser side				Ozone water irrigated side			
	
	GI	PI	GBI	MCP-1	GI	PI	GBI	MCP-1
GI								
Correlation	1.000	0.111	0.022	0.528**	1.000	0.271	−0.292	−0.063
p-value	.	0.560	0.908	0.003	.	0.147	0.117	0.740
PI								
Correlation	0.111	1.000	−0.379*	0.269	0.271	1.000	−0.008	0.219
p-value	0.560	.	0.039	0.151	0.147	.	0.966	0.245
GBI								
Correlation	0.022	−0.379*	1.000	−0.070	−0.292	−0.008	1.000	0.335
p-value	0.908	0.039	.	0.713	0.117	0.966	.	0.070
MCP-1								
correlation	0.528**	0.269	−0.070	1.000	−0.063	0.219	0.335	1.000
p-value	0.003	0.151	0.713	.	0.740	0.245	0.070	.

* Significant;

** Highly Significant; A statistically significant positive correlation between the concurrent changes of clinical parameters and MCP-1 values has been seen on the laser side during the study period

For all the clinical parameters and MCP-1 levels derived from GCF, a significant reduction was observed with both subgingival irrigation with ozonated water and laser irradiation during all time points. Not only clinical parameters were affected, but both ozonated water and laser irradiation also yielded a significant reduction in MCP-1 enzyme activities from baseline to day 28. In addition, there was a statistically significant difference among experimental and control sides for all the parameters evaluated at all the study time points, which was not present during the baseline measurements (Table 1).

## 4. Discussion

The present study aimed to compare the efficacy of subgingival ozone water irrigation and low-level laser therapy and clinical parameters to determine their clinical efficacy in mitigating gingivitis in patients receiving fixed orthodontic therapy. The results suggest that low-level laser irradiation as an adjunct treatment during orthodontic maneuvers yielded better results in reducing inflammation and gingivitis in comparison to ozone water irrigation.

One of the oft-stated objectives of orthodontic treatment is to promote better dental health and prolonging the life of a full dentition. However, if orthodontic treatment or orthodontic appliances cause significant and permanent periodontal pathology it would be difficult to justify orthodontic treatment [2]. To this end, preventing gingival inflammation from severe pathology is of major concern. There are different methods that proved successful in controlling gingival inflammation, especially in orthodontic patients. Gingival inflammation was assessed by evaluating clinical parameters such as GI, PI, and GBI. The MCP-1 marker in GCF activity may increase around teeth wearing orthodontic appliances even if they do not undergo orthodontic movement, possibly as a consequence of gingival inflammation produced by the presence of plaque retentive disposition of the used device [10]. Results from this report showed that clinical parameters such as GI, PI and GBI were reduced after subgingival irrigation with 0.01 mg/L ozonated water and after applying laser irradiation in a split-mouth model. Our results were in line with the findings reported by Dhingra and Vandana, which evaluated the clinical effects of single subgingival irrigation with ozonated water on gingival inflammation in 15 orthodontic patients and found a significant reduction in clinical parameters for gingival inflammation after subgingival irrigation with ozonated water [9]. Similar findings were reported by others showing consistent improvement in gingival inflammation on irrigation with ozone jet in patients undergoing fixed orthodontic treatment [11,19]. In other studies investigators observed significant improvements in clinical parameters when evaluating the efficacy of ozone irrigation in groups of chronic periodontitis patients [7,8,20]. Our findings were in accordance with the study conducted by Ren *et al.*, who demonstrated a significant decrease in clinical parameters after laser irradiation in orthodontic patients [21]. Similarly, in other studies, the efficacy of conservative treatment with laser therapy in chronic periodontitis patients was also successful with a significant reduction in clinical parameter values associated with gingival inflammation [15,16,22-24].

In the present study, mean MCP-1 values were reduced significantly after subgingival irrigation with 0.01 mg/L ozonated water and on the application of laser during the study period. Our results were in agreement with Sandra *et al.*, who conducted a study in orthodontic patients to evaluate gingival inflammation after subgingival irrigation with ozonated water in orthodontic patients and to correlate clinical parameters such as PI, GI, GI, and PPD with MCP-1 activity in GCF. Their study showed a statistically significant decrease in gingival inflammation and MCP-1 levels in GCF associated with ozone water irrigation [10].

The present study lasted for a period of 28 days, which is not sufficient to evaluate the long-term effect of single laser application in reducing gingival inflammation in orthodontic patients where the expected treatment duration is for 18 – 24 months. Although the carry-across effect is a potential consequence in split-mouth designs, our study was conducted on the maxillary arch to minimize the impact because of strict eligibility criteria in participant recruitment and though population sample is low it served as an adequate sample size. Looking into the future it is expected that specific laser technologies will become more available and serve an essential component of contemporary orthodontic practice where they can be used as an adjunct or alternatives to traditional approaches in managing gingival inflammation. The results of this study are based on short-term observations, however in long-term follow-up studies it is anticipated that further verification of laser therapy would further merit its anti-inflammatory biostimulation in specific patient groups. There is a need for a greater number of randomized controlled prospective clinical trials in order to determine the effect of low-level laser therapy in reducing gingival inflammation. Parameters such as frequency and duration of laser application are important when considering a therapeutic application with good response to the therapy indication.

Clinical parameters and the mean concentration of MCP-1 in GCF were reduced more significantly using diode laser irradiation when compared to treatment with ozonated water irrigation in patients receiving orthodontic therapy with fixed orthodontic appliance. The duration the present study was 28 days and is a time interval that coincides with an orthodontic patient’s monthly orthodontic treatment appointments. We would recommend applying low-level laser therapy as an adjunct to control gingival inflammation in patients undergoing orthodontic treatment. Nevertheless, it is crucial that each patient practice a strict personal oral hygiene routine. Due to the different interactions of lasers with biological tissues it is advised that knowledge of their correct use is of extreme importance for clinical success.

### Conflict of Interest

The authors declare no conflicts of interest.
